# Clinical evaluation of prophylactic abdominal aortic balloon occlusion in patients with placenta accreta: a systematic review and meta-analysis

**DOI:** 10.1186/s12884-019-2175-0

**Published:** 2019-01-15

**Authors:** Li Chen, Xiaodan Wang, Hengyu Wang, Qin Li, Nan Shan, Hongbo Qi

**Affiliations:** 1 The Department of Obstetrics, The First Affiliated Hospital of Chongqing Medical University, Chongqing, 400016 China; 2 State Key Laboratory of Maternal and Fetal Medicine of Chongqing Municipality, Chongqing Medical University, Chongqing, 400016 China; 3 International Collaborative Laboratory of Reproduction and Development of Chinese Ministry of Education, Chongqing Medical University, Chongqing, 400016 China

**Keywords:** **P**lacenta accreta, Abdominal aortic balloon occlusion, Cesarean section

## Abstract

**Background:**

Severe obstetric hemorrhage caused by placenta accreta results in significant maternal morbidity and mortality. As a new technology, abdominal aortic balloon occlusion (AABO) is becoming an important treatment for patients with placenta accreta. To evaluate the safety and efficacy of AABO, we conducted a systematic review and meta-analysis of previous studies.

**Methods:**

We used a three-check subset including placenta accreta (placenta previa, percreta, increta, etc.), balloon, and aortic (aortas, aorta, etc.) to form a retrieval formula and searched in MEDLINE, EMBASE, the Cochrane Library, clinicaltrials.gov and Web of Science. All articles regarding placenta previa or placenta accreta and including the use of abdominal aortic balloon occlusion were included in our screening. Two researchers selected articles and extracted data independently. Finally, the Newcastle-Ottawa Quality Assessment Scale was used for quality assessments.

**Results:**

We retrieved 776 articles and eventually included 11 clinical studies. Meta-analysis showed that AABO significantly reduced the blood loss volume (MD − 1480 ml, 95% CI -1806 to − 1154 ml, *P* < 0.001) and blood transfusion volume (MD − 1125 ml, 95% CI -1264 to − 987 ml, *P* < 0.001). Similarly, obvious reductions in the hysterectomy rate (OR 0.30, 95% CI 0.19 to 0.48, *P* < 0.001), hospitalization duration (MD − 1.35 days, 95% CI -2.40 to − 0.31 days, *P* = 0.01), and operative time (MD − 29.23 min, 95% CI -46.04 to − 12.42 min, *P* < 0.001) were observed in the AABO group.

**Conclusion:**

The prophylactic use of AABO in patients with placenta accreta is safe and effective.

## Introduction

The mechanism of morbidly adherent placenta (MAP) is still unknown. MAP is a pathophysiological change in which the placental villus invades the myometrium of the uterus due to dysplasia of the decidua basalis or a traumatic endocardial defect [1]. A case-control study showed that the incidence of MAP was 1.7/10000 among all delivering women, and that the proportion was as high as 577/10000 among pregnant women with placenta previa and a history of previous cesarean section [2]. As the global rate of cesarean section increases [3], especially in China, with a rate of close to 50% and even higher in some cities, the incidence of MAP is also increasing. Placenta accreta causes massive hemorrhage, disseminated intravascular coagulation (DIC), and liver and kidney damage and can even require hysterectomy. Placenta percreta not only easily causes injury to the bladder and ureters but also increases patients’ susceptibility to developing life-threatening hemorrhage. The average blood loss volume in patients with placenta increta or percreta has been reported to be 3000 ml, and this volume exceeds 5000 ml in 20% of patients and even 10,000 ml in 10% of patients [4, 5].

Previously, hysterectomy was the main approach implemented when uncontrollable hemorrhage occurred during cesarean surgery in patients with placenta accreta**.** However, hysterectomy permanently affects fertility, and some women may want another child. In recent decades, some intravascular interventional therapies have been used in obstetrics to reduce intraoperative bleeding and decrease the rate of hysterectomy, including uterine artery embolization, common iliac artery balloon occlusion, and bilateral internal iliac artery balloon occlusion [6–8]. Due to the abundant collateral circulation of the uterus, the efficacy of the above methods is limited, and these methods may even cause complications, such as hematoma, thrombosis, arterial injury, and bladder injury.

The prophylactic use of abdominal aortic balloon occlusion (AABO) in patients with placenta accreta is a new strategy that has been applied in obstetrics in recent years [9, 10]. Interventional doctors insert a balloon catheter into the lower segment of the abdominal aorta beneath the opening of the renal arteries through the right femoral artery. After the fetus is delivered and the cord is clamped, the balloon is immediately inflated to block the uterine blood supply temporarily to minimize hemorrhage when the surgeon manually removes the placenta and sutures the uterine incision. Although some related articles have been published, little is known about the efficacy of this method as these studies report contradictions that may be related to clinical heterogeneity. Given this lack of knowledge, we collected related articles and performed a systematic review and meta-analysis to evaluate the safety and efficacy of AABO.

## Materials and methods

### Search strategy

We retrieved literature from MEDLINE, EMBASE, the Cochrane Library, clinicaltrials.gov and Web of Science and used MeSH and text words to generate a three-check subset including placenta accreta (placenta previa, percreta, increta, etc.), balloon, and aortic (aortas, aorta, etc.). The retrieval formula for this research was generated using “AND” to connect the three subsets. The search included no specific language restrictions and was completed by two individuals.

### Study selection and data extraction

The population investigated included pregnant women with placenta accreta who delivered by cesarean section. Those in the experimental group underwent AABO before surgery, while those in the control group underwent direct cesarean section without balloon occlusion. Individual patients with contraindications to AABO or other intervention methods were excluded in each article. The outcomes obtained were intraoperative hemorrhage, blood transfusion volume, hysterectomy, postoperative hospitalization duration, operative time, neonatal status and complications.

The study was divided into three steps. In the first step, two individuals independently reviewed the titles and abstracts of identified articles and then obtained the full texts of the articles that seemed relevant to this study. In the second step, these individuals carefully read the original texts and selected articles for inclusion or exclusion. In the last step, the latest or most complete versions of duplicated articles were selected, and any disagreements were arbitrated by a third party. The quality assessment was completed by two researchers using the Newcastle-Ottawa Quality Assessment Scale.

### Statistical analysis

Fixed-effects models were used to analyze the hysterectomy outcome in each study because the corresponding data showed good homogeneity. The remaining data were analyzed using random-effects models because of their obvious heterogeneity. The heterogeneity of exposure factors across these studies was assessed by forest plots and quantified by the I^2^ statistic. Funnel plots were adopted to evaluate publication bias. Finally, Review Manager 5.3 was used for the statistical analysis.

## Results

**Fig. 1:**
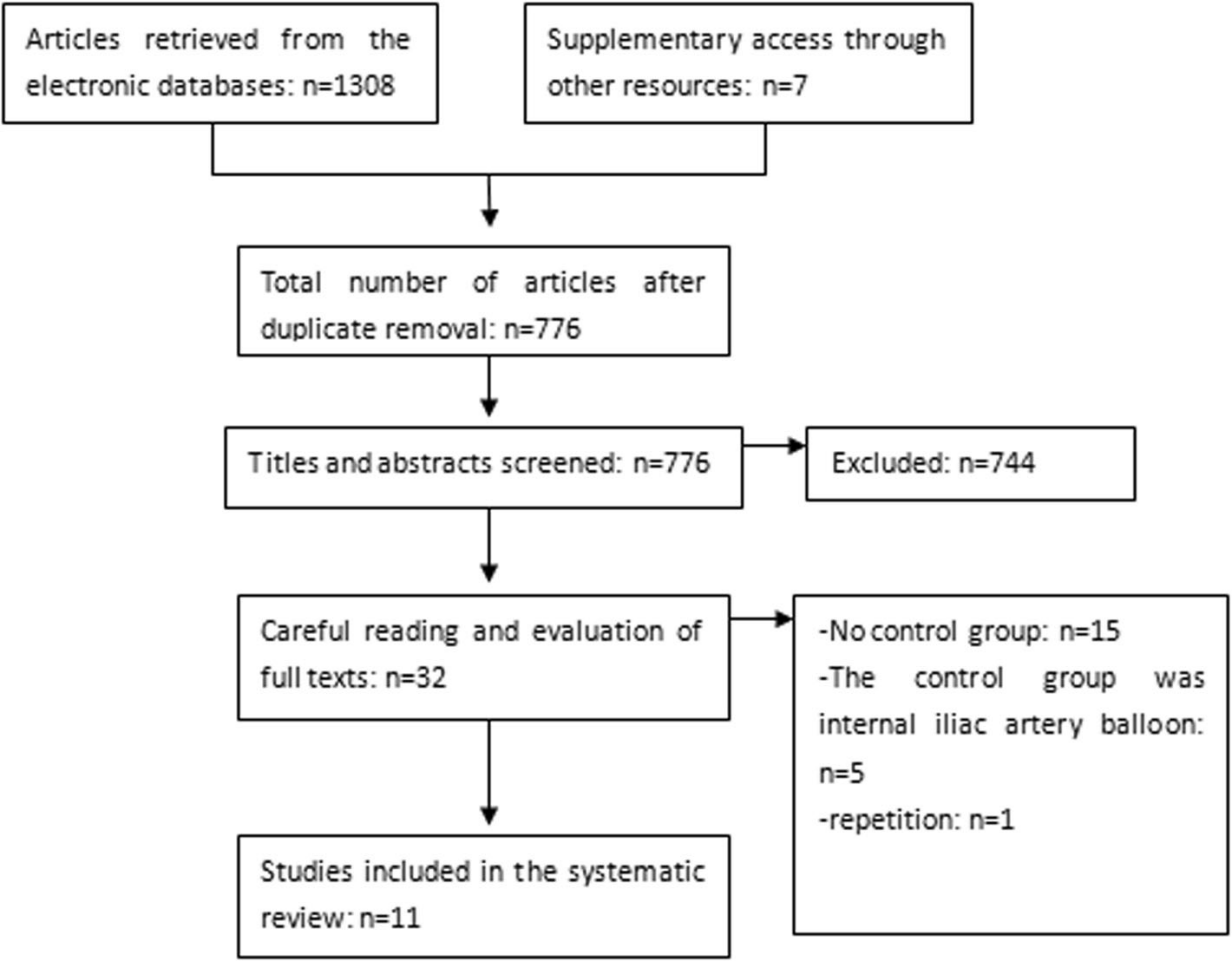
Study selection process for the meta-analysis and the inclusion and exclusion criteria

A total of 776 articles were identified in the electronic databases and other resources using the retrieval formula, 744 of which were excluded based on the titles and abstracts. The full texts of the remaining 32 studies were read, and 11 articles were finally included. All the included articles described observational studies with the target population (patients with placenta accreta) and whether the patients selected AABO after doctors explained each treatment option in detail (exposure factor) [11–21]. Twenty-one studies were excluded as summarized in Fig. 1**.**

All 11 included studies were published between 2012 and 2018 and contained a total of 731 patients. Among these patients, 463 chose to undergo AABO, while the other 268 patients did not (NO-AABO). The corresponding Newcastle-Ottawa Quality Assessment results are presented in Table 1.

**Table 1:** Appraisal of methodological quality (Newcastle-Ottawa Scale)

Reference	Case-cohort representative	Selection of non-exposed control	Ascertainment of exposure	Outcome negative at start	Comparability by design	Comparability by analysis	Outcome assessment	Duration of follow-up	Adequacy of follow-up	Score
Panici et al., 2012 [15]	√	√	√	√	√	×	√	√	√	8
Qiu et al., 2015 [16]	√	√	√	√	√	×	√	√	√	8
Cui et al., 2016 [12]	√	√	√	√	√	×	√	√	√	8
Chen et al.,2016 [11]	√	√	√	√	×	×	√	√	√	7
Wu et al., 2016 [19]	√	√	√	√	×	√	√	√	√	8
Duan et al., 2016 [13]	√	√	√	√	√	√	√	√	√	9
Sun et al., 2018 [17]	√	√	√	√	√	×	√	√	√	8
Xie et al., 2017 [20]	√	√	√	√	√	×	√	√	√	8
Gong et al.,, 2017 [14]	√	√	√	√	√	×	√	√	√	8
Wang et al., 2017 [18]	√	√	√	√	√	×	√	√	√	8
Wei et al., 2018 [21]	√	√	√	√	×	×	√	√	√	7

### Meta-analysis

**Fig. 2:**
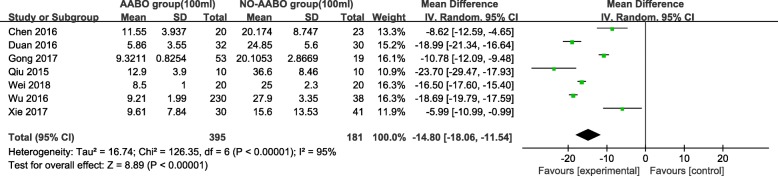
Forest plot of studies for blood loss volume (100 ml)

**Fig. 3:**
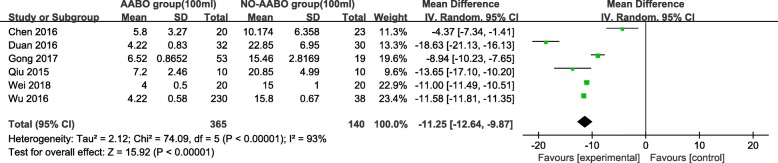
Forest plot of studies for blood transfusion volume (100 ml)

**Fig. 4:**
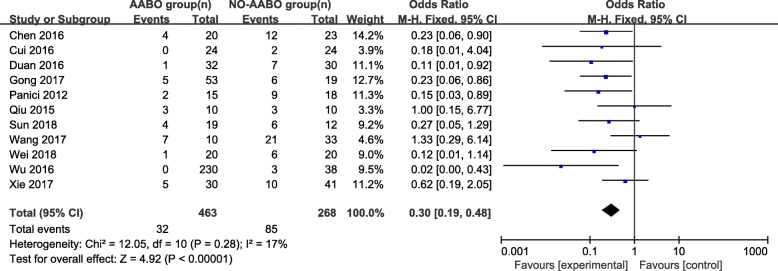
Forest plot of studies for the hysterectomy rate

**Fig. 5:**
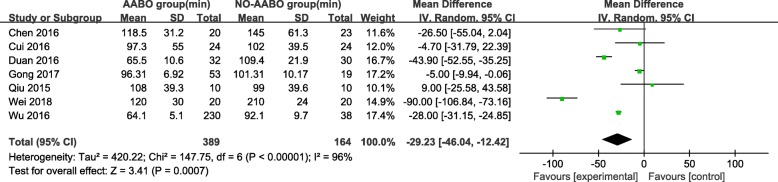
Forest plot of studies for operative time (min)

**Fig. 6:**
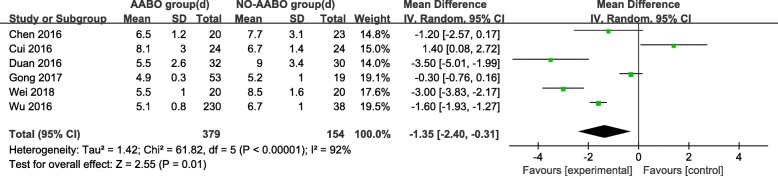
Forest plot of studies for postoperative hospitalization duration (days)

Overall, 7 articles compared intraoperative hemorrhage between the AABO and NO-AABO groups. The prophylactic use of AABO before surgery significantly reduced the blood loss volume compared with no AABO (MD -1480 ml, 95% CI -1806 to − 1154 ml, *P* < 0.001) (Fig. 2). The blood transfusion volume was reported in 6 studies; overall, patients who underwent AABO required fewer PRBC unit transfusions than those who did not undergo AABO (MD -1125 ml, 95% CI -1264 to − 987 ml, *P* < 0.001) (Fig. 3). The difference in the hysterectomy rate between patients who underwent AABO and those who did not was compared in all 11 studies. Similarly, obvious differences were found between the two groups (OR 0.30, 95% CI 0.19 to 0.48, *P* < 0.001) (Fig. 4). Seven articles listed the operative time, with a clearly reduced time in the AABO group compared with that in the NO-AABO group (MD − 29.23 min, 95% CI -46.04 to − 12.42 min, *P* < 0.001) (Fig. 5). Meta-analysis of the 6 studies that reported the number of postoperative hospitalization days as an outcome showed a significant reduction in the postoperative hospitalization duration with AABO (MD -1.35 days, 95% CI -2.40 to − 0.31 days, *P* = 0.01) (Fig. 6).

Four studies reported no obvious differences in Apgar scores between the AABO and control groups, with a mean score of greater than 8. Regarding intraoperative or postoperative maternal complications, among 4 articles, the numbers of disseminated intravascular coagulation (DIC) events were 0 and 8 in the AABO and control groups, respectively. In addition, a total of 3 cases of hematoma at the puncture site and 5 cases of venous thrombus were reported in the AABO group, with a balloon-related morbidity rate of 1.7% (*n* = 8/463). These patients were all cured during hospitalization. However, in addition to the 8 cases of DIC, 12 cases of hemorrhagic shock due to uncontrollable hemorrhage resulting from manual removal of the placenta were reported in the control groups**.**

### Evaluation of publication bias

**Fig. 7:**
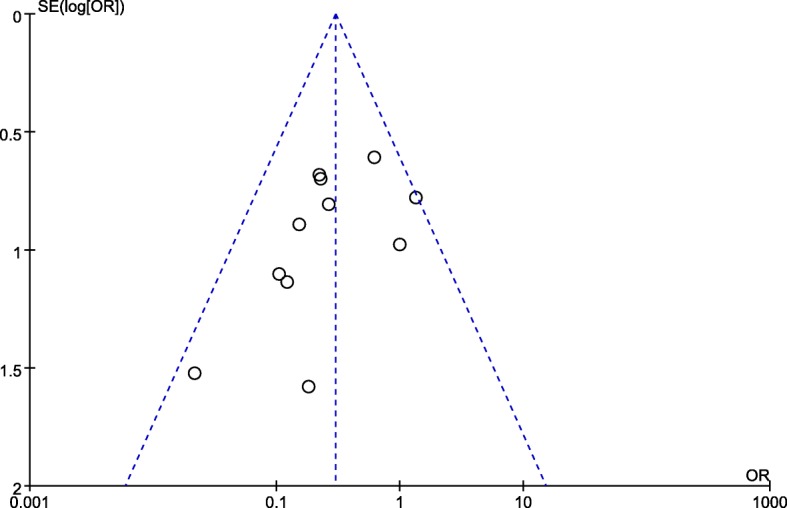
Funnel plot of the publication bias for the hysterectomy rate

Funnel plots for articles evaluating AABO versus NO-AABO in terms of the hysterectomy rate showed asymmetry on visual inspection, with gaps suggesting that few studies with negative results have been published (Fig. 7).

## Discussion

This study is the first systematic review and meta-analysis to evaluate the safety and efficacy of AABO during manual removal of the placenta during cesarean section in patients with placenta accreta. The conclusion of this study is that compared with no AABO, the prophylactic use of AABO has a beneficial effect by decreasing hemorrhaging and blood transfusion requirements during or after surgery. Patients treated with AABO had lower hysterectomy and DIC rates. Additionally, the operative time and postoperative hospitalization duration were shorter in the AABO group than those in the NO-AABO group.

The main complications of AABO were thrombus and hematoma at the puncture site, with an incidence rate of 1.7% (*n* = 8/463). These patients all recovered spontaneously or with thrombolytic therapy. Compared with no AABO, AABO prevented some severe complications caused by uncontrollable hemorrhage after manual removal of the implanted placenta, while 8 cases of DIC and 12 cases of hemorrhagic shock were reported in the NO-AABO group.

The weaknesses of our study are mainly related to the heterogeneity of the results. We read the original articles again in an attempt to identify the source of the heterogeneity from among the features of the population, inconsistencies in the exposure factors and differences in the methodologies; however, no significant differences were found among these studies. Therefore, we speculate that the heterogeneity may originate at the levels of the surgeon, patient condition assessments, and blood loss volume estimations. In addition, although most articles mentioned that the patients included in their studies were complicated with placenta increta or percreta, they did not describe the specific area and depth of placental implantation in detail, which may be one of the sources of heterogeneity.

Some scholars have expressed concerns that X-rays cause fetal damage. The International Commission on Radiological Protection (ICRP) suggests that the fetal teratogenic risk does not increase when the radiation dose is less than 100 mGy [22]. In this study, all articles reported fetal radiation exposure doses of less than 10 mGy. Three other articles compared AABO and bilateral internal iliac artery balloon occlusion in terms of the radiation dose and concluded that AABO resulted in a lower fetal radiation dose [23–25], indicating that AABO is safer for fetuses than other endovascular interventional treatments in patients with placenta accreta. In our study, no puerperal or neonatal complications caused by radiation were reported during the follow-up period.

With the development of endovascular interventional treatments, some scholars have attempted to apply a balloon catheter to the distal abdominal aorta, bilateral common iliac artery or internal iliac artery in patients with placenta accreta to temporarily block uterine blood flow and provide adequate time for surgeons. However, because of the extensive collateral circulation between the arteries in the pelvis [26], the hemostatic effect of iliac artery balloon occlusion, embolization or ligation is limited. Ramoni et al. [27] reported 4 cases of placenta accreta accompanied by postpartum hemorrhage after bilateral uterine artery ligation due to collateral circulation. Shrivastava et al. [28] reported no significant reduction in the blood loss or transfusion volume in patients with placenta accreta when bilateral internal iliac artery balloon occlusion was applied.

For AABO, the balloon is placed below the renal arteries to block most of the blood supply to the pelvic cavity, thereby exerting a better hemostatic effect. Yang et al. [23] and Wang et al. [25] reported significant reductions in the blood loss volume and the number of blood transfusions with AABO versus bilateral internal iliac artery occlusion. A systematic review compared the efficacy of balloon occlusion of the internal iliac arteries, abdominal aorta, uterine artery, and common iliac arteries and showed that AABO resulted in less blood loss and a lower rate of hysterectomy than the other methods [29].

No uniform standard for the total duration of balloon occlusion is available. Masamoto et al. [9] reported a case of a patient with placenta percreta treated with 80 min of continuous occlusion during cesarean section, with no obvious intraoperative or postoperative complications. In our study, 5 articles reported the occlusion time. The mean time ranged from 18 min to 36.95 min in these articles, with a maximum occlusion time of 80 min. However, occlusion was not continuous, allowing intermittent recovery of the blood supply. No limb necrosis, ischemia-reperfusion injury, functional renal impairment, or spinal cord or peripheral nerve injury was observed postoperatively during the follow-up period.

## Conclusion

Placenta accreta caused severe obstetric hemorrhage and results in significant maternal morbidity and mortality. Abdominal aortic balloon occlusion (AABO), as a new technology, is becoming an important treatment for patients with placenta accrete. It has a beneficial effect by decreasing hemorrhaging and blood transfusion requirements during or after surgery and reduces the hysterectomy and DIC rates. It is safe and effective and should be promoted in patients with placenta accreta.
